# Initial results of the INSPIRE clinical trial—investigating radiation dosimetry for differentiated thyroid cancer patients

**DOI:** 10.3389/fnume.2023.964478

**Published:** 2023-05-15

**Authors:** Jan Taprogge, Carla Abreu, Lenka Vávrová, Lily Carnegie-Peake, Dominic Rushforth, Paul Gape, Jonathan Gear, Iain Murray, Kee H. Wong, Kate Newbold, Siraj Yusuf, Glenn Flux

**Affiliations:** ^1^ Joint Department of Physics, Royal Marsden NHSFT, Sutton, United Kingdom; ^2^ Radioisotope Physics, Institute of Cancer Research, London, United Kingdom; ^3^ Thyroid Unit, Royal Marsden NHSFT, Sutton, United Kingdom; ^4^ Department of Nuclear Medicine and PET/CT, Royal Marsden NHSFT, Sutton, United Kingdom

**Keywords:** multi-centre trial, dosimetry, radioiodine, differentiated thyroid cancer, residual thyroid tissue

## Abstract

**Introduction:**

The optimal strategy for differentiated thyroid cancer (DTC) patients treated with radioiodine (RAI) following thyroidectomy remains controversial. Multi-centre clinical studies are essential to identify strategies to improve patient outcomes while minimising treatment-induced toxicity.

**Materials and Methods:**

The INSPIRE clinical trial (ClinicalTrials.gov Identifier: NCT04391244) aims to investigate patient-specific dosimetry for DTC patients and to determine the range of absorbed doses delivered to target and non-target tissues and their relationship with treatment outcome and toxicity.

**Results:**

We report here initial results of the first 30 patients enrolled onto the INSPIRE trial. A large range of absorbed doses are observed for both thyroid remnants and salivary glands, with median values of 4.8 Gy (Range 0.2 – 242 Gy) and 0.3 Gy (Range 0.1 to 1.7 Gy), respectively.

**Discussion:**

The preliminary study results are encouraging and could help to improve our understanding of absorbed doses to thyroid remnants and normal organs following RAI therapy. Such knowledge could potentially enable patient-specific treatment planning with improved clinical outcomes and quality-of-life of patients.

## 1. Introduction

More than 80 years after the initial use of radioiodine (RAI), controversy remains regarding the optimal treatment regimen for thyroid cancer patients. A group of experts from the American Thyroid Association (ATA), the European Association of Nuclear Medicine (EANM), the Society of Nuclear Medicine and Molecular Imaging (SNMMI) and the European Thyroid Association (ETA) published a consensus paper (1) highlighting several issues that need addressing. Considerable variability remains between centres in Europe with respect to the decision-making process following thyroidectomy (2).

The decision to treat with RAI following thyroidectomy and the level of activity to administer should be based on the risk-benefit ratio. The benefit of RAI therapy, especially for low-risk differentiated thyroid cancer (DTC) patients, remains controversial (3–5). Leboulleux et al. showed in a prospective, randomised, phase 3 trial (ESTIMABL2) of patients with low-risk DTC [T1(m) N0 M0] that surveillance is non-inferior to RAI therapy for event-free survival at 3 years (6). A similar study, IoN, is currently investigating this question with incorporation of a higher-risk group (up to T3 and N1a disease) (7). Nevertheless, further work may also be required with respect to larger patient groups and long follow-ups (8). Two randomised trials (HiLo and ESTIMABL1) reported similar post-ablation success at 6–9 months and recurrence rates in patients with well-differentiated thyroid cancer when comparing 1.1 GBq and 3.7 GBq (9–11). Further studies are studying prognostic markers to predict ablation success (12). Ablation success has been hypothesised to be dependent on the absorbed dose delivered to any residual thyroid tissue rather than the RAI administered activity (13–16), with several studies showing a large range of absorbed doses for empiric activities (13–21).

Controversy with respect to optimal treatment is in part due to the lack of robust evidence in the literature concerning the potential risks of RAI treatment. Salivary disorders are a potential side effect of RAI and have been reported as early as weeks or months after treatment (22, 23). These findings have been supported by systematic reviews and meta-analysis with respect to salivary and lacrimal gland dysfunction. However, due to major methodological differences between studies, the reported incidence of these disorders ranges from 16 to 72% (24). Long-term side effects such as second primary malignancy (SPM) have been investigated in several epidemiological studies. Increased risk of SPM in patients with DTC has been shown in a meta-analysis (25, 26) but evidence is usually classed as low quality and the effect has shown to be small with a relative risk ranging from 1.14 to 1.84 of RAI vs. no RAI (27). Retrospective epidemiological studies aiming to address the risk of SPM after RAI treatment for DTC have produced contradicting results (28–30) and this remains a matter of debate (31).

Patient-specific dosimetry could potentially lead to patient-tailored treatment planning and may be used to assess radiation risks. Interpretation of European Council Directive 2013/59/Euratom varies between countries and centres (32–35). Large multi-centre multi-national prospective clinical studies are required to address the controversies including the risk from RAI and to assess the relationship between the absorbed dose delivered to targets and treatment outcome (36). Examples of multi-centre clinical studies which have included dosimetry for radioiodine are SEL-I-METRY (EudraCT No 2015-002269-47) (37, 38) and MEDIRAD (39), a project funded by European Horizon 2020 that investigated the implications of medical low-dose radiation exposure through a multi-centre multi-national prospective study to assess the radiation doses from RAI therapy in 100 DTC patients using quantitative imaging (40).

INSPIRE (Investigating National Solutions for Personalised Iodine-131 Radiation Exposure, ClinicalTrials.gov Identifier: NCT04391244) follows on from MEDIRAD to further investigate the range of absorbed doses to target and non-target tissues DTC patients and aims to assess the correlation between the absorbed doses and clinical outcome and/or toxicities.

We report here the initial results of the INSPIRE study, with focus on the range of absorbed doses delivered to target (residual thyroid tissue) and non-target (salivary glands and whole-body) tissues.

## 2. Materials and methods

INSPIRE is currently a single centre, prospective observational study with approval to expand to a multi-centre study in the United Kingdom. The overarching hypothesis is that treatment outcome in molecular radiotherapy is dependent on the absorbed doses delivered rather than on the radioactivity administered. With a target recruitment number of 50 patients, the primary endpoint is to establish the range of absorbed doses and associated uncertainties delivered to thyroid remnants, residual disease and healthy organs from Na[^131^I]I. The study was approved by the East Midlands—Nottingham 1 Research Ethics Committee (20/EM/0022) and the institutional review board at the Royal Marsden Hospital. All patients provided written informed consent prior to registration.

### 2.1. Patient inclusion criteria

Inclusion criteria include patients with histologically proven DTC treated with total thyroidectomy or staged surgery (hemithyroidectomy followed by completion thyroidectomy) who are 18 years or older and had their first treatment with RAI. Patients were excluded from the study if they had a prior diagnostic RAI scan, external beam radiotherapy or systematic chemotherapy within 6 weeks of treatment.

### 2.2. Radioiodine administration

Patients were administered either 1.1 or 3.7 GBq of Na[^131^I]I according to local protocols. Administration was performed following stimulation using recombinant human thyrotropin (rhTSH). Patient preparation included a low iodine diet but no specific salivary gland secretion stimulation protocol (41).

### 2.3. Data collection for quantitative imaging and dosimetry

Imaging systems in participating centres were prepared for quantitative imaging to allow collation of data (36, 37). Two dosimetry gamma camera scanning schedules were developed to account for initial COVID-19 restrictions and patient preferences. For schedule 1, a single standard-of-care SPECT-CT scan is acquired according to local protocol post-RAI administration. Schedule 2 includes the standard-of-care scan and a minimum of two additional SPECT scans between 6 and 168 h post-RAI administration.

For both scanning schedules, a minimum of 3 whole-body (WB) retention measurements were performed per day during the patient's stay in hospital, approximately every 2–6 h, according to local standard of care procedures. At each external measurement time point, the quantified level of radioactivity in the whole body was recorded using a ceiling-mounted radiation detector above the patient's bed.

Patients were followed-up at their standard-of-care clinic visits with routine blood tests including thyroid function test and thyroglobulin. These data are not reported here as the follow-up data collection is currently ongoing.

### 2.4. Thyroid remnant and salivary gland dosimetry calculations

SPECT imaging datasets were reconstructed with CT attenuation and Monte-Carlo scatter corrections. Images were quantified using system volume calibration factors as described previously (40). Dosimetry calculations were performed using in-house dosimetry software developed at the RMH using Slicer3D (42). Time-integrated activity (TIA) was determined using single or multiple time-point fitting using a single exponential decay function as applicable.

For single time-point dosimetry, assumed effective half-lives of T_1/2_ = 68 h, 9.3 and 8.6 h were used for the thyroid remnant (21), parotid and submandibular glands (43), respectively.

Organs were outlined using segmentation tools available in Slicer3D. The thyroid remnant and salivary glands were segmented. All other organs showed little physiological uptake and were assumed to have activity levels too low to be quantified accurately. The thyroid remnant was outlined on the SPECT image using thresholding with a relative threshold value of 10%. Salivary glands were outlined on the CT to obtain the volume and reproduced on SPECT scans using thresholding to obtain the retention activity.

The absorbed dose to the voxel with maximum uptake (13) was calculated for the thyroid remnant, while the mean absorbed dose to salivary glands were determined using dose kernels taking into account the electron contribution to the absorbed dose only.

### 2.5. Whole-body dosimetry calculations

Whole-body absorbed doses were calculated from the whole-body retention measurements. A multi-exponential decay function was fitted to the data to obtain the AUC allowing for up to 4 different exponential decay phases. Whole-body absorbed doses were calculated using the Medical Internal Radiation Dose (MIRD) (44) formalism with a mass-adjusted S-factor as described by Buckley et al. (45).

### 2.6. Statistical analysis

The D'Agostino & Pearson test was used to test for normality of the distributions of absorbed doses and absorbed doses per unit of administered activity for each tissue. The results of all normality tests indicated that the null hypothesis must be rejected in all cases (*p* < 0.05 for all distributions) and the conclusion was drawn that the data are not normally distributed. All absorbed dose results are, therefore, reported as median (range). The Mann-Whitney test was employed to assess if thyroid remnant, salivary gland and WB absorbed doses per unit of administered activity were significantly different between patients treated with 1.1 and 3.7 GBq, respectively.

All statistical tests were exploratory, and testing was performed at the two-sided 5% significance level. All statistical analysis was performed using GraphPad Prism version 9.3.1 or later for Windows (GraphPad Software, San Diego, California USA).

## 3. Results

The preliminary analysis includes the first 30 DTC patients (3) recruited at a single centre (Royal Marsden Hospital). A summary of patient characteristics is provided in Table 1. Nineteen patients participated with scanning schedule 2 with two additional SPECT scans between 20 and 72 h post-administration. Eleven patients participated with a single standard-of-care SPECT-CT scan due to COVID-19 restrictions and patient preferences. Post-therapy SPECT-CT scans did not reveal any metastases in these patients.

**Table 1:** Characteristics of the patients (*n* = 30).

Characteristic	
Age—year (Mean ± Standard Deviation)	44.8 ± 15.9
Female—*N* (%)	22 (73.3)
**Histological subtype—*N* (%)**	
Papillary	19 (63.3)
Follicular	11 (36.7)
**Primary tumour and node stage—*N* (%)**	
T1N0	7 (23.3)
T1N1a	2 (6.7)
T1N1b	2 (6.7)
T2N0	6 (20.0)
T3N0	7 (23.3)
T3N1a	3 (10.0)
T3N1b	2 (6.7)
T3Nx	1 (3.3)
**Prescribed RAI activity—*N* (%)**	
1,100 MBq	12 (40.0)
3,700 MBq	18 (60.0)

### 3.1. Dosimetry results for thyroid remnants and salivary glands

**Figure 1:**
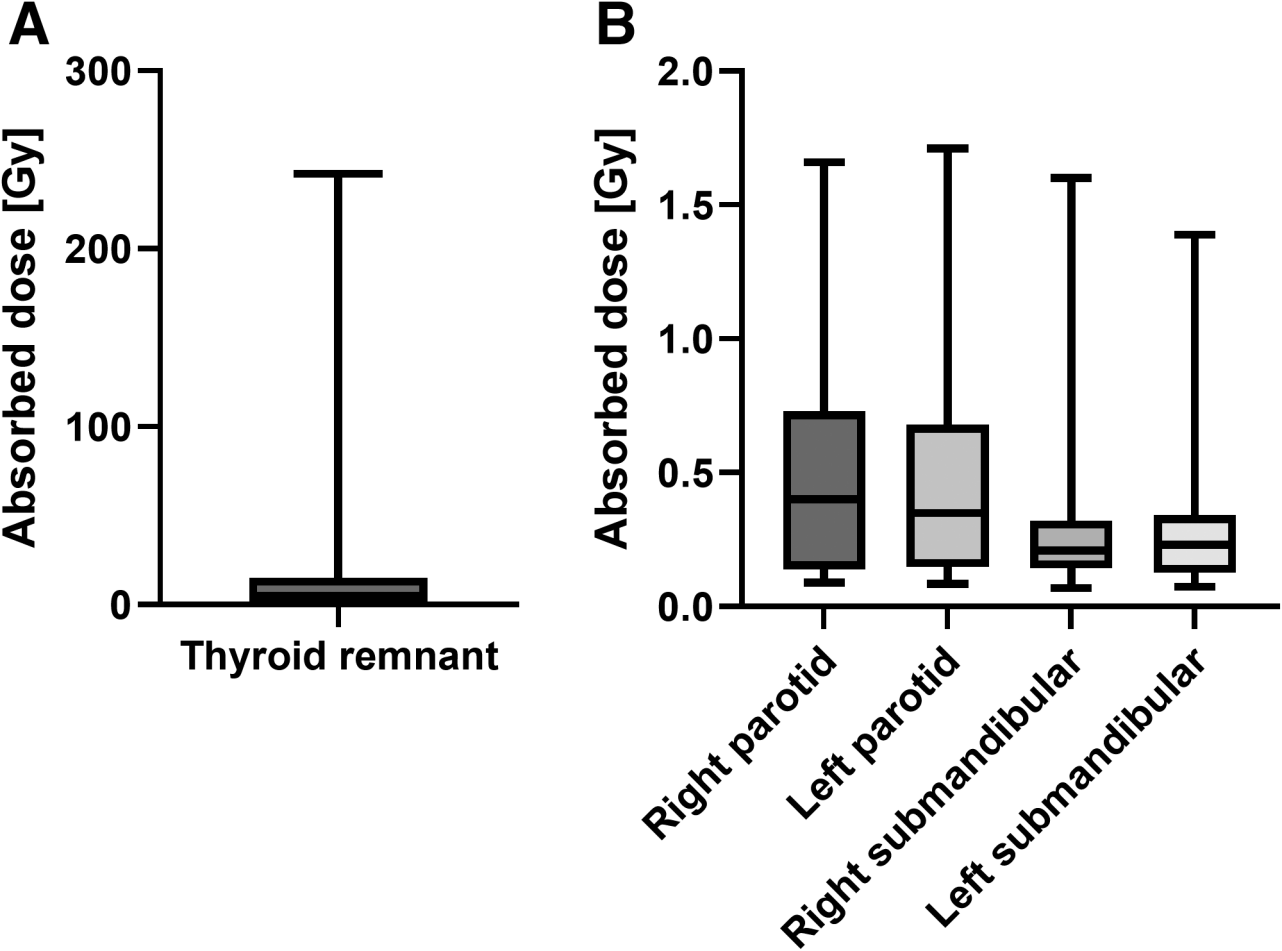
Range of (**A**) thyroid remnant maximum-voxel absorbed doses and (**B**) salivary glands absorbed doses.

**Figure 2:**
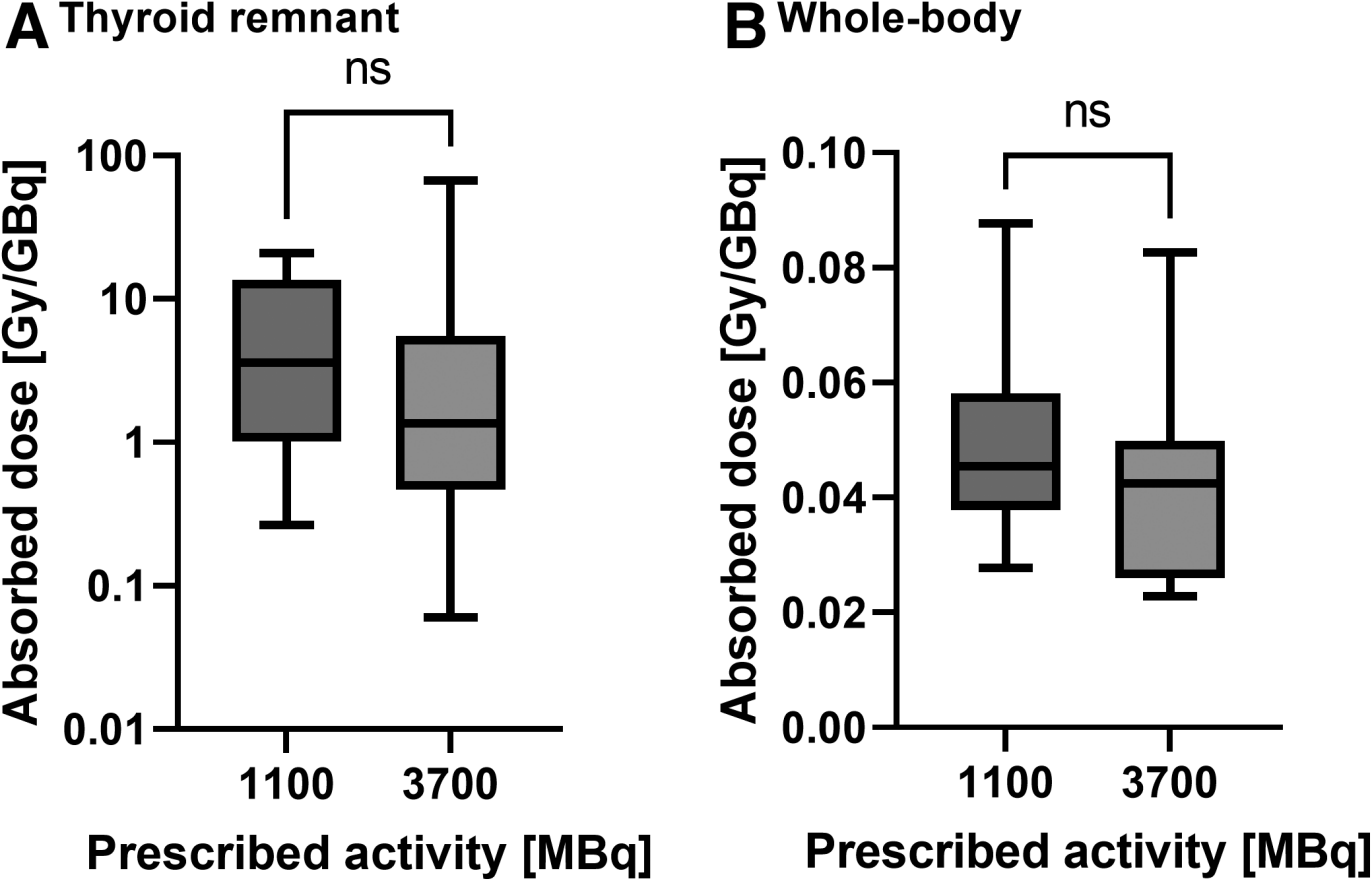
Comparison of the absorbed doses per unit of administered activity for patients prescribed 1.1 and 3.7 GBq, respectively, for (**A**) the thyroid remnant and (**B**) the whole-body. The results of the Mann-Whitney test are indicated above each comparison with **“**ns” = non-significant (*p*-value > 0.05).

**Figure 3:**
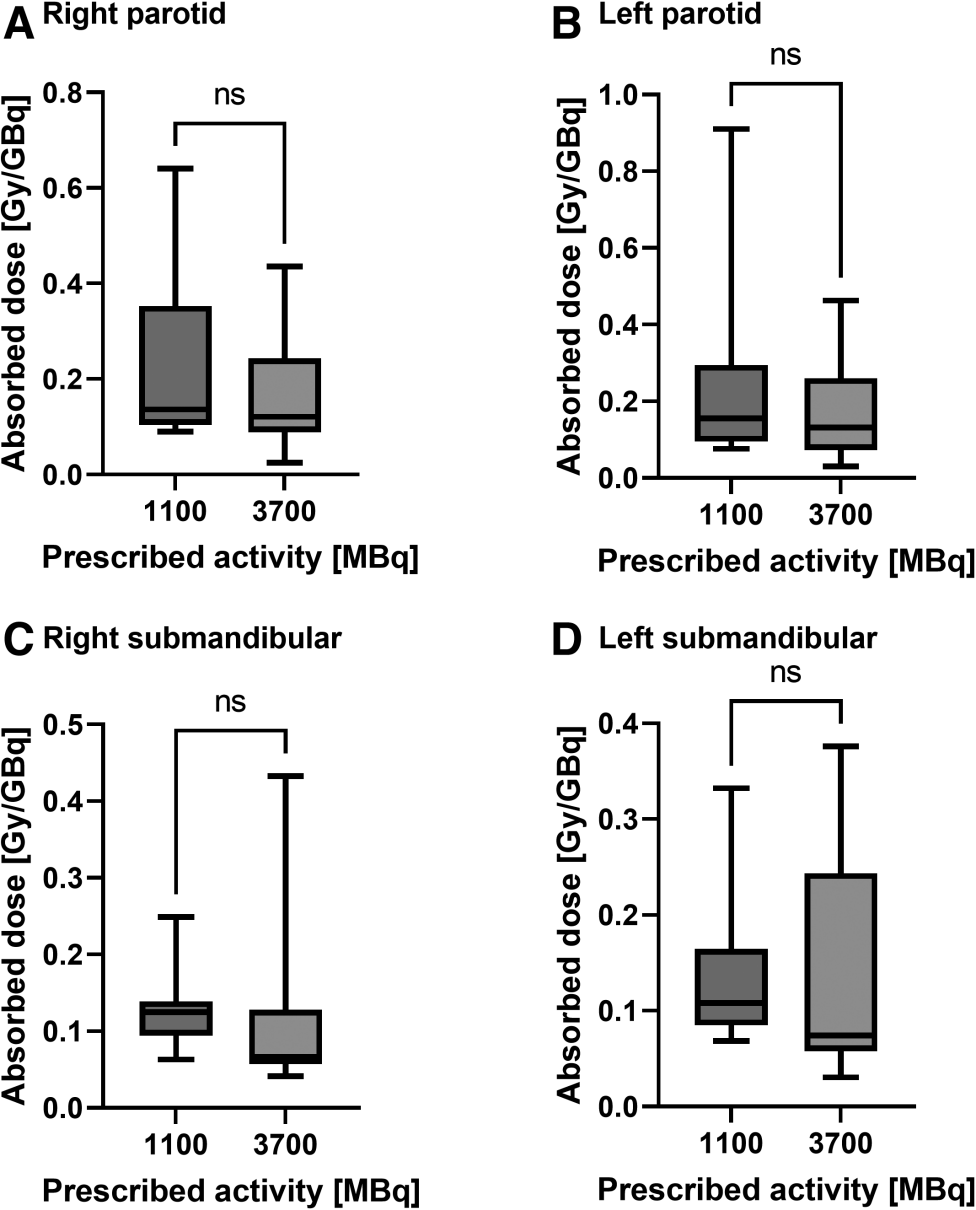
Comparison of the absorbed doses per unit of administered activity for patients prescribed 1.1 and 3.7 GBq, respectively, for (**A**) the right parotid, (**B**) the left parotid, (**C**) the right submandibular and (**D**) the left submandibular gland. The results of the Mann-Whitney test are indicated above each comparison with **“**ns” = non-significant (*p*-value > 0.05).

Table 2 and Figure 1 show the absorbed doses estimated for the thyroid remnant and salivary glands. A wide range of absorbed doses (0.2 to 242 Gy) was observed for the thyroid remnant. Figures 2A, 3 show the comparison of absorbed doses per unit of administered activity for the thyroid remnant and salivary glands for patients treated with 1.1 and 3.7 GBq, respectively. The results of the Mann-Whitney tests between the absorbed doses per unit of administered activity for patients who received 1.1 and 3.7 GBq showed that the difference was non-significant in all cases. The *p*-values of the tests for thyroid remnant, right parotid, left parotid, right submandibular and left submandibular glands were 0.26, 0.67, 0.39, 0.07 and 0.13, respectively. This could potentially indicate that absorbed doses scale linearly with administered activity.

**Table 2:** Range of absorbed doses to salivary glands and thyroid remnant.

Organs [Gy]	Median	Minimum	Maximum
Thyroid remnant*	4.8	0.2	242.0
Parotid right**	0.4	0.1	1.7
Parotid left**	0.4	0.1	1.7
Submandibular right**	0.2	0.1	1.6
Submandibular left**	0.2	0.1	1.4

* Absorbed dose to voxel with maximum uptake.

** Mean absorbed dose to outlined VOI.

Patients scanned according to schedule 2 were found to have median effective half-lives of 42.3 (16.1–99.9) hours, 12.9 (6.7–23.6) hours and 11.9 (7.0–85.3) hours in thyroid remnant, parotid glands and submandibular glands, respectively.

### 3.2. Dosimetry results for whole-body

**Figure 4:**
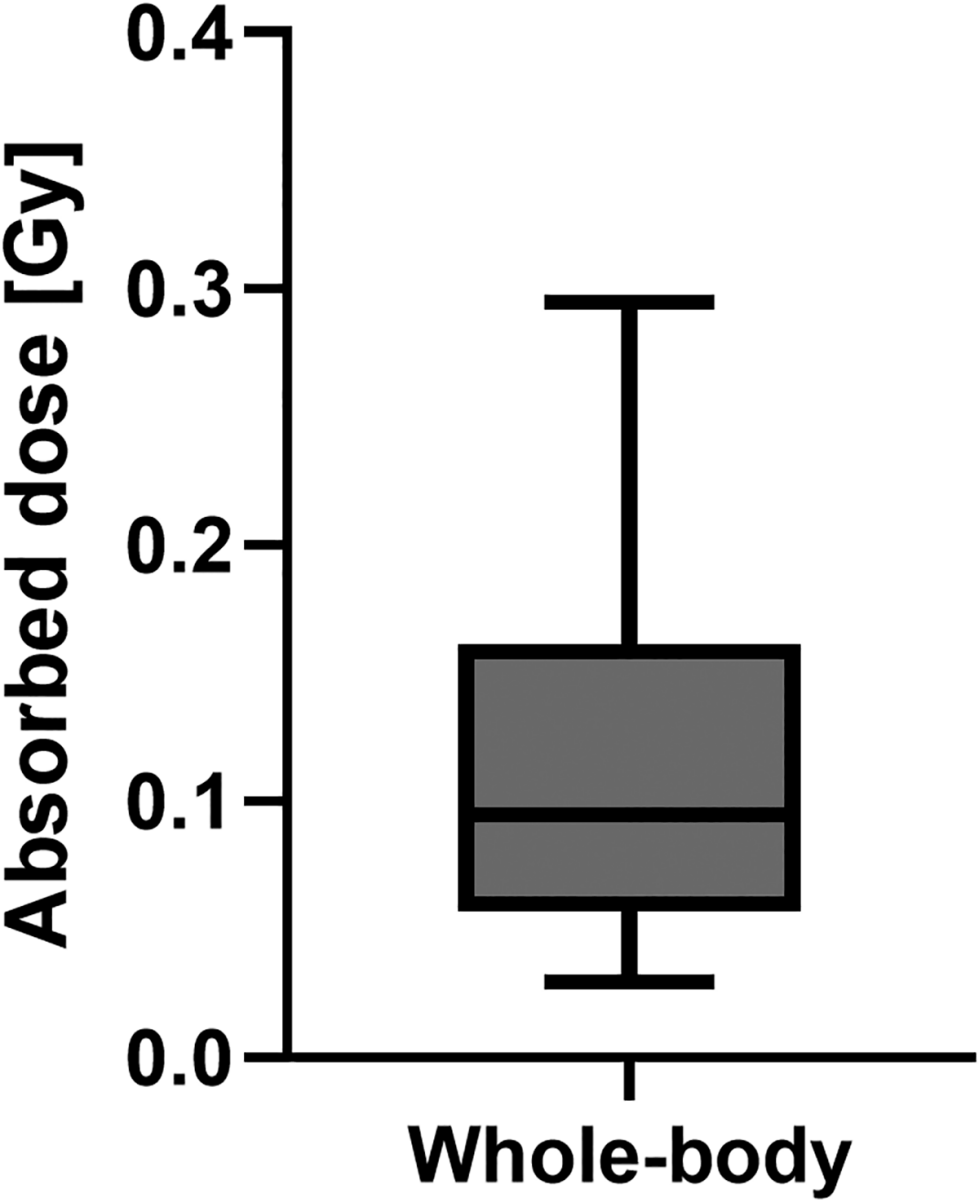
Whole-body absorbed doses obtained from the whole-body retention measurements.

The median whole-body absorbed dose was 0.10 Gy (Range 0.03**–**0.29 Gy). The range of whole-body absorbed doses is illustrated in Figure 4. Figure 2B shows the comparison of whole-body absorbed doses per unit of administered activity for patients treated with 1.1 and 3.7 GBq respectively. The results of the Mann-Whitney test between the whole-body absorbed doses per unit of administered activity for patients who received 1.1 and 3.7 GBq showed that the difference was non-significant (*p* = 0.25). This also indicates that absorbed doses scale with administered activity.

## 4. Discussion

A large range of absorbed doses are observed for both thyroid remnants and salivary glands which implies the need for and potential benefit of personalised treatment planning in this patient cohort. The results presented here are from a single centre, but previous studies (37, 40) have demonstrated that dosimetry in a multi-centre setting is feasible. Standardisation of quantitative imaging and dosimetry methodologies across centres or a central dosimetry hub for processing is essential to be able to collate results from centres and investigate dose-response relationships. An important finding of these preliminary results is that absorbed doses to target and non-target tissues scale with the administered activity as no significant difference could be found between absorbed doses per unit of administered activity for patients treated with 1.1 and 3.7 GBq, respectively.

The majority of maximum-voxel thyroid remnant absorbed doses were below 50 Gy, which is lower than values reported in a previous publication by Flux et al. (13) in the same centre. Possible explanations for this observation are advances in diagnostic imaging, allowing for improved visualization of small thyroid remnants, and the improvement in surgery due to centralisation of patient care to high volume centres in the United Kingdom with differences in the amount of remaining thyroid tissue following surgery. Significant progress has also been made with respect to image reconstruction, processing and dosimetry calculations which explains the large differences of absorbed doses when compared to the study by Maxon et al. (46) that proposed an absorbed dose threshold of 300 Gy for the successful ablation of thyroid remnants, significantly higher than the absorbed doses calculated in the present study. A direct comparison of absorbed doses in this contemporary study with the historical studies is therefore challenging and absorbed dose thresholds are in need of re-evaluation. The large range of absorbed doses indicates that the majority of patients are either under- or over-treated. Further work is therefore required to achieve standardisation of methodologies and to establish dose thresholds in large-scale multi-centre clinical studies which could be used for personalised treatment planning in this cohort.

The range of absorbed doses to salivary glands is much lower than the mean *gland* absorbed dose limits used in external beam radiotherapy (EBRT), which recommend to spare parotid glands to less than 20 to 26 Gy (47, 48). It is worth noting that these limits are for fractionated radiotherapy to mitigate grade 3 xerostomia while the salivary gland toxicities observed following RAI are usually of grade 1 or 2. Furthermore, due to radiobiological factors such as relative biological effectiveness, heterogeneous dose distribution and dose rate effects, absorbed doses delivered cannot be directly compared to EBRT. In the multi-centre phase of the study, INSPIRE will collect salivary gland toxicity data up to 24 months following therapy using Common Terminology Criteria for Adverse Events (CTCAE) version 5.0 criteria that will enable investigation into the relationship between the absorbed dose to salivary glands and treatment-induced toxicity. The median absorbed dose values per unit of administered activity obtained here of 0.2 (Range 0.1–0.9) mGy/MBq and 0.1 (0.1–0.9) mGy/MBq for parotid and submandibular glands, respectively, can be compared with the values of 0.2 (0.1–0.3) mGy/MBq and 0.5 (0.2–1.2) mGy/MBq provided by Jentzen et al. (49). Despite the low absorbed dose, salivary gland toxicity is well recognised in this patient population who generally expect a good quality-of-life (QoL), as has been reported in the literature (24). Jentzen et al. (49) proposed that an inhomogeneous distribution of RAI in human salivary glands could be a possible explanation which would lead to a very heterogenous dose distribution. Further work on this is required and a possibility to overcome the clear limitations of the spatial resolution of the imaging system would be the use of pharmacokinetic modelling, as has been performed by Taprogge et al. (50) for the example of ^223^Ra.

The measured half-lives for thyroid remnants and salivary glands are in agreement with values published in the literature (21, 43). Nevertheless, the large range of observed half-lives is of importance when considering the possibility of single-time point dosimetry in this patient cohort, as discussed by Gustafsson et al. (51).

Limitations of the present study include that the results presented here include only low- and intermediate-risk patients without the presence of metastases and in a single centre. The aim is to expand the study to multiple centres and include high-risk patients and to perform lesional-dosimetry to establish the range of absorbed doses and to assess the relationship between absorbed doses and outcome in these patients.

## 5. Conclusions

These early study results define pragmatic methodologies to improve understanding of absorbed doses to thyroid remnants and normal organs following RAI therapy. An enhanced knowledge of the impact of this treatment should enable superior clinical outcomes whilst minimising treatment-induced toxicities.

## Data Availability

The original contributions presented in the study are included in the article, further inquiries can be directed to the corresponding author.
